# Neutrophil extracellular trap-associated risk index for predicting outcomes and response to Wnt signaling inhibitors in triple-negative breast cancer

**DOI:** 10.1038/s41598-024-54888-y

**Published:** 2024-02-20

**Authors:** Zhidong Huang, Jinhui Wang, Bo Sun, Mengyang Qi, Shuang Gao, Hong Liu

**Affiliations:** 1 The Second Surgical Department of Breast Cancer, Tianjin Medical University Cancer Institute & Hospital, National Clinical Research Center for Cancer, Tianjin, China; 2 Tianjin’s Clinical Research Center for Cancer, Tianjin, China; 3 Key Laboratory of Breast Cancer Prevention and Therapy, Tianjin Medical University, Ministry of Education, Tianjin, China

**Keywords:** Cancer, Cancer therapy, Tumour biomarkers, Computational biology and bioinformatics

## Abstract

Triple-negative breast cancer (TNBC) is a type of breast cancer with poor prognosis, which is prone to distant metastasis and therapy resistance. The presence of neutrophil extracellular traps (NETs) contributes to the progression of breast cancer and is an efficient predictor of TNBC. We obtained the bulk and single-cell RNA sequencing data from public databases. Firstly, we identified five NET-related genes and constructed NET-related subgroups. Then, we constructed a risk index with three pivotal genes based on the differentially expressed genes between subgroups. Patients in the high-risk group had worse prognosis, clinicopathological features, and therapy response than low-risk group. Functional enrichment analysis revealed that the low-risk group was enriched in Wnt signaling pathway, and surprisingly, the drug sensitivity prediction showed that Wnt signaling pathway inhibitors had higher drug sensitivity in the low-risk group. Finally, verification experiments in vitro based on MDA-MB-231 and BT-549 cells showed that tumor cells with low-risk scores had less migration, invasion, and proliferative abilities and high drug sensitivity to Wnt signaling pathway inhibitors. In this study, multi-omics analysis revealed that genes associated with NETs may influence the occurrence, progression, and treatment of TNBC. Moreover, the bioinformatics analysis and cell experiments demonstrated that the risk index could predict the population of TNBC likely to benefit from treatment with Wnt signaling pathway inhibitors.

## Introduction

Female breast cancer (BC) accounted for 24.2% of all incident cancer cases (2.1 million), based on the global cancer statistics from 2020^1^. Triple-negative breast cancer (TNBC), which is negative for the expression of estrogen receptor, progesterone receptor, and human epidermal growth factor receptor 2, accounts for about 15–20% of all breast cancers^2^. Compared with Luminal and human epidermal growth factor receptor 2 enriched subtypes, TNBC is subject to poor prognosis, lack of therapeutic targets, high rate of recurrence and metastasis, and chemotherapy resistance^3^. With progress in high-throughput sequencing, single-cell sequencing, spatial transcriptome sequencing, and computational biology technology, several novel potential biomarkers at the genetic and epigenetic levels have been discovered from tissues or peripheral blood samples^4–6^, providing a basis for new approaches for the diagnosis and treatment of BC.

As the most abundant type of granulocytes or leukocytes, neutrophils are indispensable effector cells in the process of innate immunity, and their effects are primarily exerted through phagocytosis, granulation, and release of neutrophil extracellular traps (NETs)^7^. Neutrophils can support tumor proliferation and metastasis by adjusting the death and migration of tumor cells, immunoreaction, and angiogenesis through NETosis, that is, the NET formation process of neutrophils^8^. Active neutrophils release proteins and DNA–histone complexes that make up NETs^9^. Furthermore, NETs promote tumor progression and metastasis. For example, two types of NET-related proteases, neutrophil elastase and matrix metalloproteinase 9 can cleave laminin, an integrin α3β1 activation epitope. This leads to FAK/ERK/MLCK/YAP signaling in tumor cells, reactivating dormant cancer cells^10^. Yang et al. found that the transmembrane protein CCDC25 of BC cells recognizes DNA from extracellular NET components to activate the ILK–β-parvin pathway to improve the motor capacity of cancer cells^11^. Patients with BC and colon cancer who develop liver metastases have high levels of NET-related DNA, which acts as a chemotactic factor to promote the liver metastasis of BC. In the early stages of BC, serum NETs are crucial for predicting liver metastases.

Tumor in an individual develops and progresses through a series of steps, ranging from tumorigenesis, metastasis, and treatment resistance. Average gene mutation frequency and gene expression is reflected by bulk RNA sequencing, which assists in better understanding of the molecular characteristics of each step of tumorigenesis by identifying sensitive biomarkers, mutations, and gene expression profiles^5,12,13^. Compared with bulk RNA sequencing, single-cell sequencing is considerably better for probing cellular and microenvironment heterogeneity at single-cell resolution. Thus, integrating clinicopathological information with single-cell sequencing data can provide more accurate biomarkers for the early diagnosis and cure of patients and explore treatment-related types or states of cancer cells)^14–16^.

Purpose and significance of the present research was to explore the features and clinical worth of NET-related genes using bioinformatics analyses and in vitro cell line experiments and provide new diagnostic markers and cure strategies, as well as predicting their survival chances. To achieve this, multi-omics data (e.g., interactome, transcriptome, proteome, and genome) were combined, and the molecular characteristics and prognostic role of NET-related genes were analyzed using bulk RNA-seq and single-cell sequencing data. Additionally, a new NET-related subtype of TNBC and a risk index linked to NETs were identified for predicting prognosis and informing TNBC treatment.

## Results

### Landscape of copy number variation and gene mutation in NET-related genes

**Figure 1:**
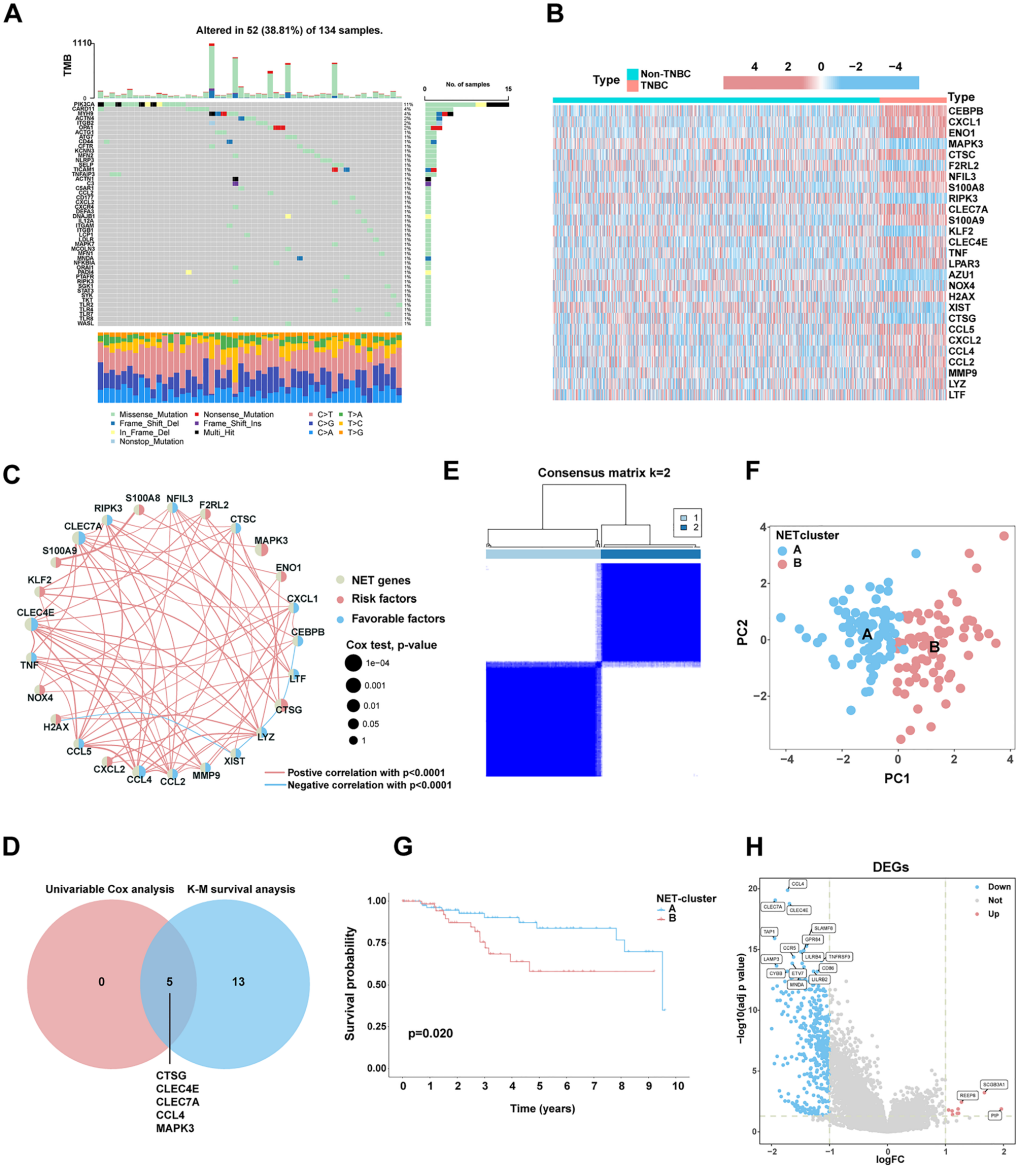
(**A**) Waterfall diagram showing the frequency and type of mutations in the NET-related genes using the R package “maftools”. (**B**) Differential expression analysis of NET-related genes in TNBC and non-TNBC tissues using Wilcoxon rank-sum tests. (**C**) Prognostic network diagram showing the co-expression relationships and the prognosis using univariate Cox regression of differentially expressed NET-related genes. (**D**) Venn diagram showing the genes in common between univariate Cox analysis and Kaplan–Meier (K–M) analysis. (**E**) Heatmap depicts consensus clustering solution (k = 2) for NET-related genes using the consensus clustering algorithm. (**F**) Principal component analysis (PCA) was performed based on the gene matrices of NET-related subtype. (**G**) K–M survival analysis comparing clusters A and B. (**H**) Volcano map shows NET-DEGs between cluster B and A.

The gain of copy number was greater than the loss of copy number in most NET-related genes. ACTG1, FCGR2B, KCNN3, MNDA, NLRP3, S100A12, S100A8, and S100A9 were more likely to show copy number gain than other genes, whereas AZU1, MFN2, LCP1, PADI4, ELANE, and PRTN3 were more likely to show copy number loss (Fig. S1). With respect to the mutation status of the NET-related genes, up to 38.81% of the samples with TNBC had NET-related gene mutations. PIK3CA had the highest gene mutation frequency of 11%. The remaining 135 genes, with a mutation rate of < 4%, were relatively conserved (Fig. 1A).

### Establishment of NET-related subtypes

A total of 27 NET-related genes were aberrantly expressed in TNBC samples based on the TCGA-TNBC cohort (Fig. 1B), which were incorporated into subsequent analyses of prognostic value. In the K–M survival analysis conducted on the TCGA-TNBC cohort, 18 NET-related genes demonstrated relevance to overall survival (OS) (Fig. S2). In the univariate Cox regression analysis, CLEC4E, CLEC7A, and CCL4 expressions were favorable factors (HR < 1), whereas positive CTSG and MAPK3 expressions were risk factors for poor prognosis (HR > 1) (Fig. 1C). The results of the K–M survival analysis and univariate Cox regression analysis were intersected to identify five prognostically related genes (Fig. 1D). Based on the five prognostic genes, TNBC patients' segmentation was based on the analysis of unsupervised consensus clustering, dividing into A and B subtypes (Fig. 1E).

### Functional enrichment and immune microenvironment analysis revealed the characteristics of NET-related subtypes

The PCA showed that the samples with TNBC are easy to tell apart based on the new NET-related subtype, which verified the effect of NET-related subtypes (Fig. 1F). K–M survival curves for clusters A and B showed that cluster A was associated with better OS (Fig. 1G). In addition, the functional enrichment analysis using GSVA indicated that immune-relevant pathways (e.g., Cytokine–cytokine receptor interactions signaling pathway, Antigen processing and presentation signaling pathway, Primary immunodeficiency, Nature killer cell-mediated cytotoxicity, T cell receptor signaling pathway, B cell receptor signaling pathway, etc.) and tumor-related pathways, such as JAK/STAT signaling pathway concentrated in cluster A (Fig. S3A). As shown in Fig. S3B, the infiltration degree of immune cell in TNBC was obviously different between A and B subtype, except for CD56^dim^ natural killer cells, eosinophils, mast cells, monocyte, and plasmacytoid dendritic cell. In general, the A subtype has higher infiltration degree.

Comparing gene expression between clusters A and B, 430 DEGs were identified (Fig. 1H). Functional enrichment analyses indicated that these genes were remarkably enriched in GO terms including leukocyte-mediated immunity, positive regulation of cell activation, external side of plasma membrane, and antigen-binding. The KEGG analysis showed that NET-DEGs were significantly enriched in immune-associated pathways (cytokine–cytokine receptor interaction, cell-adhesion molecules, chemokine signaling, Th17 cell differentiation, etc.) (Fig. S3C).

### Landscape of hub DEGs between two subtypes in single-cell expression profile

**Figure 2:**
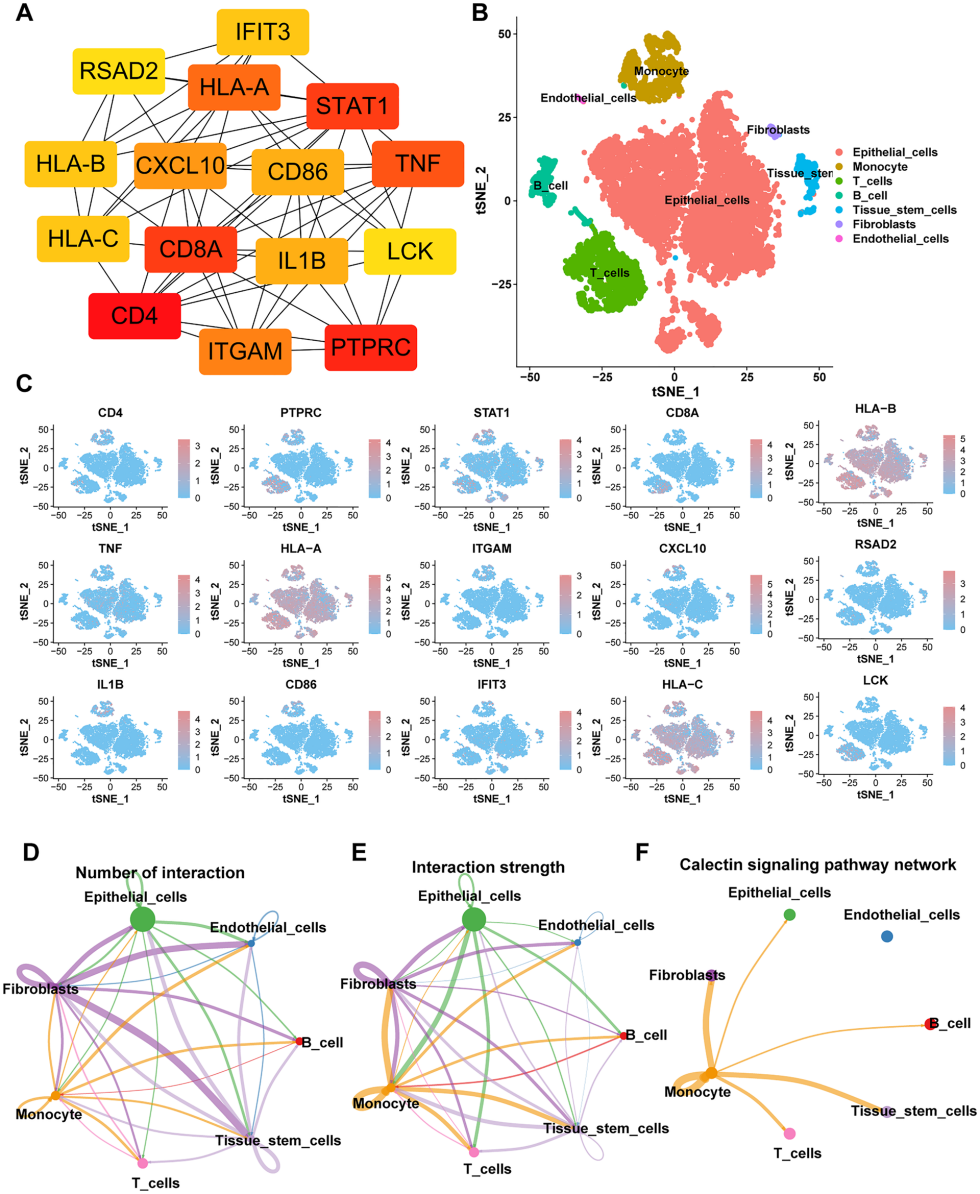
Identification and of hub NET-DEGs and the analysis of scRNA-seq data (GSE161529) (**A**) The co-expression network based on the top 15 hub NET-DEGs using the STRING online database. (**B**) tSNE plot of the unsupervised cluster analysis labeled by cell types. (**C**) The expression of hub NET-DEGs in single-cell level. (**D**) The number of cell–cell interactions between different cells in TNBC. (**E**) The cell–cell interaction strength between different cells in TNBC. (**F**) Calectin signaling pathway network in cell–cell communications.

Based on the PPI network and the degree of expression of each protein, 15 top hub DEGs were identified (Fig. 2A). Based on the scRNA-seq dataset GSE161529, 35,585 cells from four primary TNBC samples were obtained for subsequent analysis. After applying the dimension reduction method of PCA, 13 clusters were obtained. Seventeen cell subsets were obtained and visualized using tSNE analysis (Fig. 2B). Figure 2C shows the distribution of the 15 hub DEGs in single cells. Intercellular communication analysis to predict gene interactions between different cell types (Fig. 2D) showed that fibroblasts had the highest number of communications with other cell types in TNBC tissues. Figure 2E shows the communication strength among all cell types in TNBC tissues. Epithelial cells showed stronger communication with fibroblasts, monocytes, T cells, and B cells. The hub DEGs were involved in cell–cell communication between monocytes and fibroblasts, between monocytes and tissue stem cells, and among monocytes in the GALECTIN signaling pathway which was related to tumor immune evasion (Fig. 2F).

### Construction and validation of the risk index

**Figure 3:**
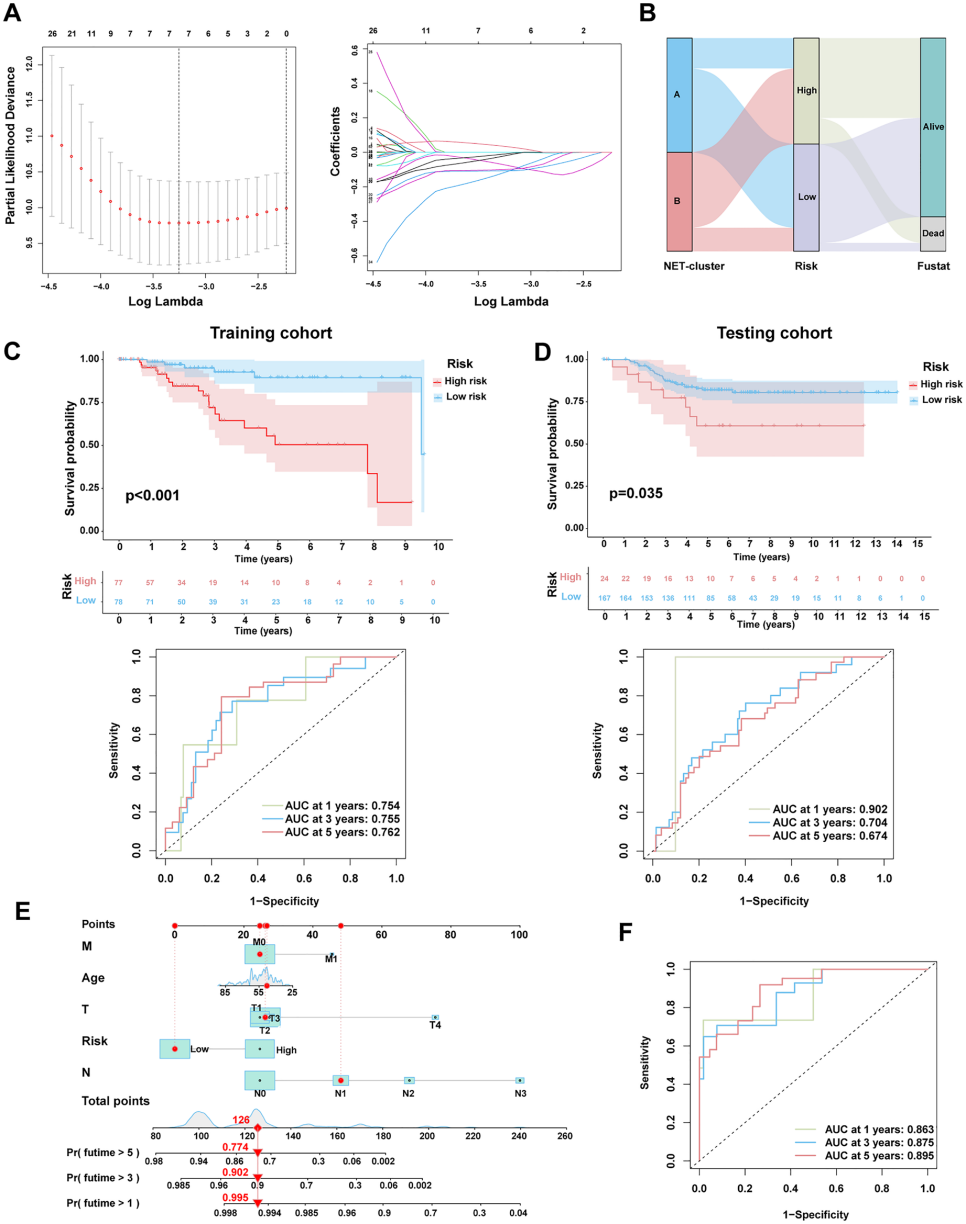
Construction of risk index and nomogram. (**A**) Partial likelihood deviance for least absolute shrinkage and selection operator (LASSO) coefficient profiles. (**B**) Sankey diagram showing the process for building the risk index and there was a statistically significant difference in death events between various risk group. (**C**) and (**D**) The time-dependent analysis of ROC and K–M survival curves were used to assess predictive accuracy of risk index and compare the difference of survival outcome between various risk group in the training cohort (TCGA-TNBC cohort) and testing cohort (GEO-TNBC cohort). (**E**) A nomogram constructed on the basis of age, T stage, N stage, M stage, and risk score generated using the R package “rms.” (**F**) The time-dependent analysis of ROC was used to assess the time-dependent accuracy of the nomogram.

A total of 62 prognostic NET-DEGs were screened via univariate Cox regression analysis (Table S3). After using TCGA-TNBC as the training cohort and GEO-TNBC as the testing cohort, we established a risk index of three genes through LASSO regression analysis (Fig. 3A). As shown in the Sankey plot, the number of death events in the high-risk group was higher than that in the low-risk group (Fig. 3B). The risk score plot, showing the relevance between the risk score and outcomes. The number of death events increased with the risk score. The heat map shows the differences in the expression of risk index-related genes between the distinct risk groups. The over-expressed gene in the high-risk group was REEP6, whereas those in low-risk group were GBP1P1, and MOXD1 (Fig. S4A,B). The time-dependent analysis of ROC and K–M survival curves suggested that the risk index had a high predictive accuracy. Consistency in results was observed across both the training and testing cohorts (Fig. 3C,D).

To better combine the risk index with clinical application we developed a nomogram based on the patient’s age, risk score, T stage, N stage, and M stage which was used to more intuitively predict the OS probability at 1, 3, and 5 years (Fig. 3E). The time-dependent accuracy of the nomogram was assessed by ROC curves (Fig. 3F).

### Clinicopathological, immune and gene mutational features of different risk groups

Apart from examining the overall survival of patients, previous studies have reported an association between NETs and the recurrence and metastasis of tumors. The K–M survival analysis revealed that higher-risk patients in the TCGA-TNBC cohort exhibited a shorter Disease-Free Interval (DFI) (Fig. S5A). Additionally, utilizing the GSE58812 dataset, we also observed that patients in the high-risk group had a shorter Metastasis-Free Survival (MFS) (Fig. S5B), which indicated that high-risk patients experience more recurrence and metastasis compared to the low-risk group.

**Figure 4:**
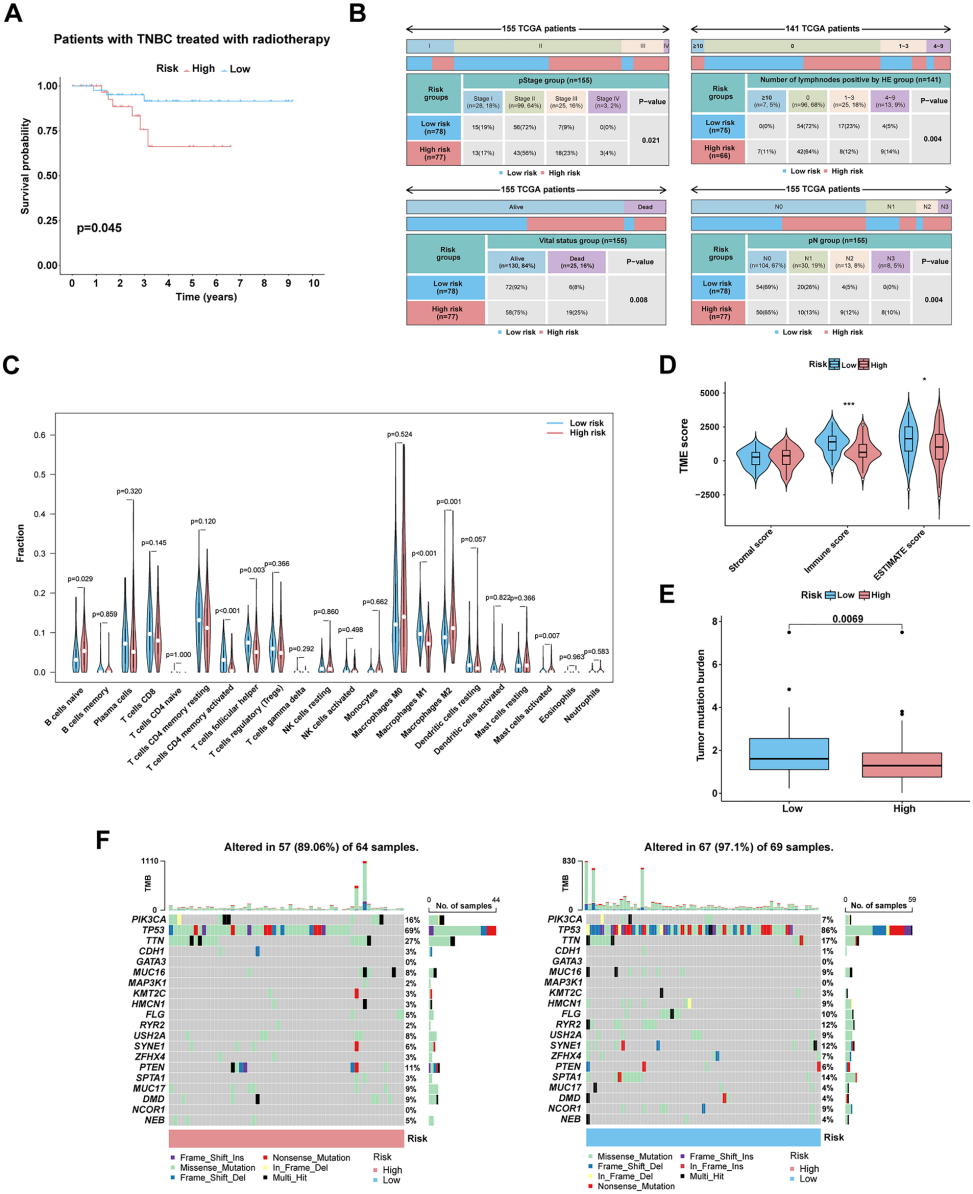
(**A**) The efficacy of radiotherapy was different in various risk groups. (**B**) The clinicopathological information and outcomes of two risk groups was compared. (**C**) The degree of immune cell infiltration of various risk groups was analyzed and compared. (**D**) “ESTIMATE” algorithm was applied to estimate the tumor stromal, immune, and estimate score of patients in disparate risk groups. (**E**) The comparison of two risk groups of TMB using Wilcoxon rank-sum tests. (**F**) Waterfall graph showing the top 20 genes in mutation frequency in disparate risk groups using the R package “maftools”.

Moreover, we analyzed the differences in clinicopathological characteristics and therapy responses of patients in various risk groups. In TNBC patients treated with radiotherapy, patients in the low-risk group had better therapeutic reaction (Figs. 4A, S5C). For chemotherapy drugs, the IC50 values of commonly used chemotherapy drugs (Cisplatin, Gemcitabine, Olaparib, Talazoparib and Vincristine) in breast cancer patients within the high-risk group were higher than those in the low-risk group (Fig. S5D). Nevertheless, patients in the high-risk group tended to show higher pathological staging, poorer survival status, more positive lymph nodes, and higher pathological N stage (Fig. 4B).

Considering that TNBC is the most immunogenic type of BC, we analyzed the role of risk scores in the tumor microenvironment. According to the CIBERSORT algorithm, we calculated the percentage abundance of 22 types of immune cells per sample to assess the relevance between the degrees of infiltration of immune cells in tumors and risk index. The results of the bar plots showed that the low-risk group had higher levels of CD4^+^ T cells, M1 macrophages, and mast cells, which have a tumor-suppressive effect, while the high-risk group had higher levels of M2 macrophages, which promote tumor growth (Fig. 4C). Furthermore, correlation between tumor microenvironment and risk scores of the various risk groups differed in the immune and estimate scores (Fig. 4D).

With respect to the correlation between the risk index and TMB, the TMB of the low-risk group was higher than high-risk group (Fig. 4E). Our findings are consistent with previous literature reporting that the higher TMB, the better OS and therapy response in TNBC. Meanwhile, TP53, PIK3CA and TTN were the top 3 frequently mutated gene in both high- and low-risk groups, and PIK3CA and TTN were more likely to be altered in the high-risk group than in low-risk group (Fig. 4F).

### Low-risk group exhibited increased drug sensitivity to Wnt signaling pathway inhibitors

**Figure 5:**
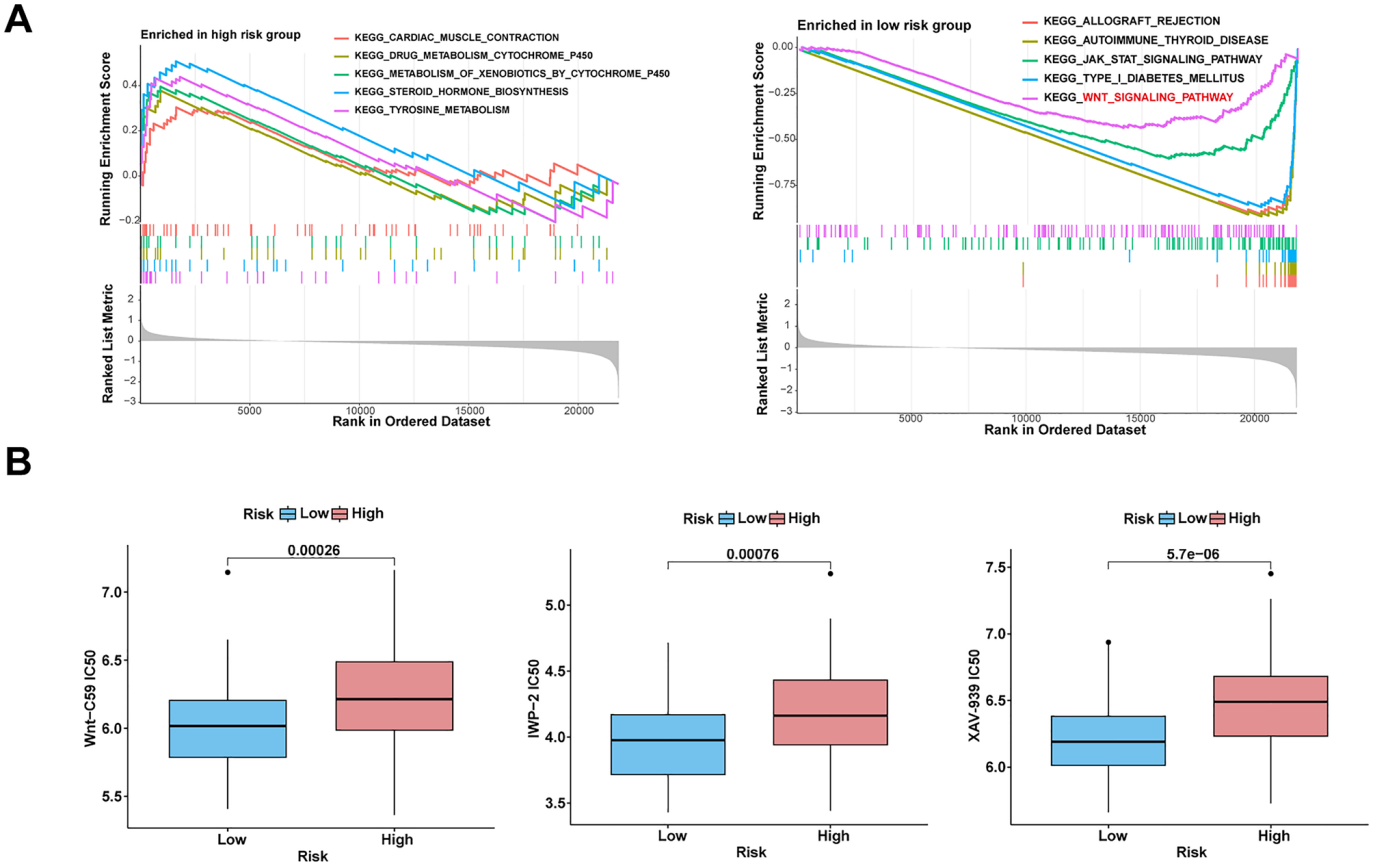
(**A**) The KEGG enrichment analysis was used to explore the potential biological functions of various groups. (**B**) Prediction and comparison of drug sensitivity to Wnt signaling pathway in various risk groups.

TNBC often has a poor prognosis because of the lack of corresponding therapeutic targets, which encouraged us to explore potential drug targets. We carried out the KEGG functional enrichment analysis. The results indicated that the high-risk group enriched in cardiac muscle contraction, drug metabolism by cytochrome p450, metabolism of xenobiotics by cytochrome p450, steroid hormone biosynthesis, and tyrosine metabolism. Also, the low-risk group was related to allograft rejection, autoimmune thyroid disease, JAK-STAT signaling pathway, Type I diabetes mellitus, and Wnt signaling pathway (Fig. 5A). It was worth noting that TNBC patients with lower risk score showed apparently higher drug sensitivity to three Wnt signaling pathway inhibitors (Wnt-C59, IWP-2, and XVA-939) (Fig. 5B). Apparently, the finding provided clues for the significance of the risk index in TNBC patients.

### TNBC cell lines with low-risk score had low malignancy and high sensitivity to Wnt signaling pathways inhibitors

**Figure 6:**
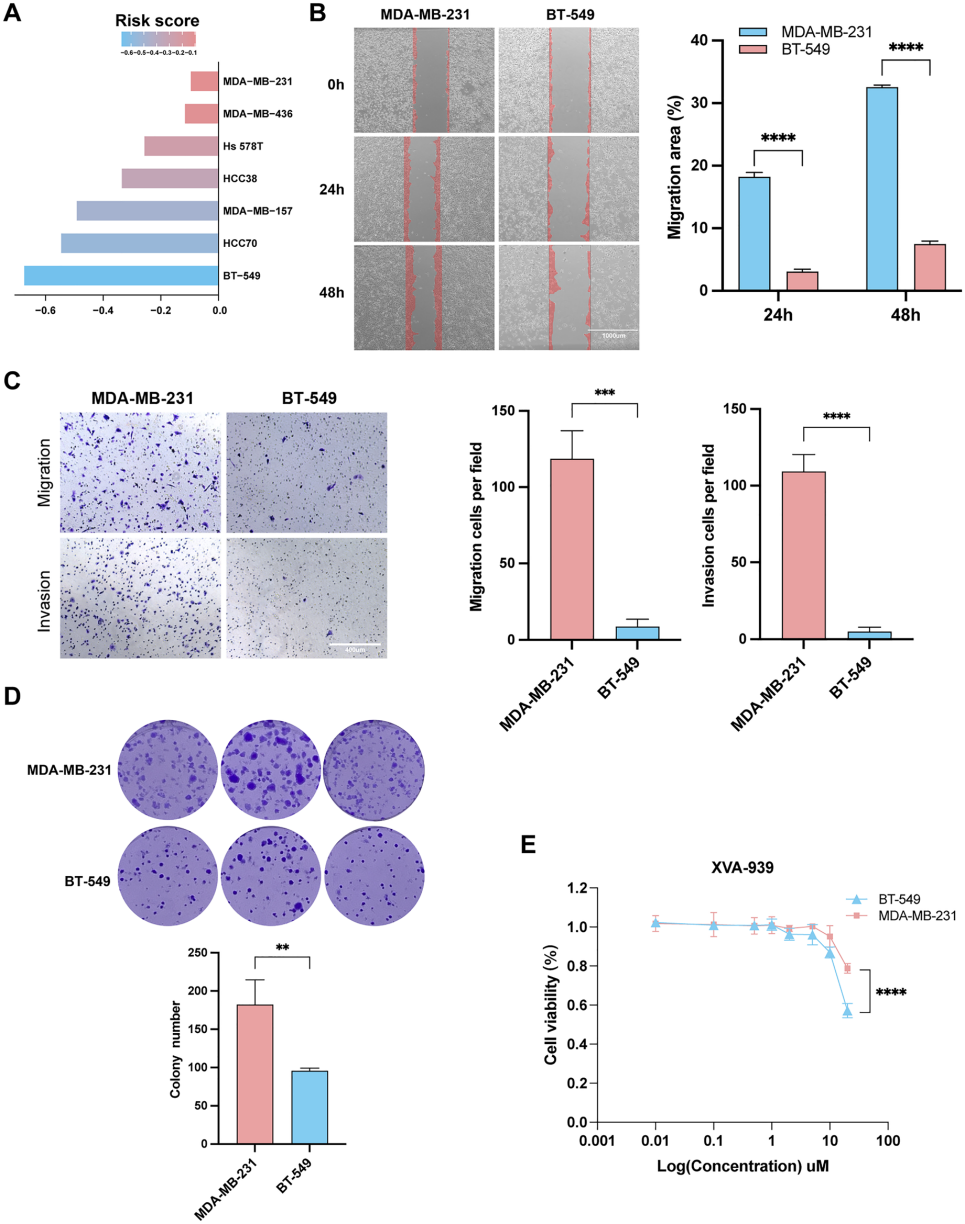
(**A**) Risk score for each TNBC cell lines calculated from the CCLE database. (**B**–**C**) The wound healing, migration, and invasion assays was performed to compare the invasion and migration ability between MDA-MB-231 and BT-549. (**D**) Colony formation assay was carried out to explore the cell proliferation ability of MDA-MB-231 and BT-549. (**E**) Cytotoxicity assay to compare the drug sensitivity of MDA-MB-231 and BT-549 to different concentrations of XVA-939.

To further validate our findings, we performed in vitro cell experiments. By the application of the CCLE database, risk scores were calculated for various TNBC cell lines. Figure 6A shows that MDA-MB-231 was the cell line with the highest risk score, while BT-549 had the lowest risk score. To validate previous studies, we performed migration, invasion, wound healing, and colony formation assays. We found that MDA-MB-231 had higher invasion, migration, and proliferation activities than BT-549 (Fig. 6B–D), consistent with the conclusion of our previous study. In addition, to verify the results of drug sensitivity prediction, we selected the Wnt signaling pathway inhibitor XVA939 with the most significant difference of IC50 between high and low risk for drug sensitivity testing. Dose‐response growth curve of XVA-939 showed that BT-549 had a higher sensitivity to XVA-939 than MDA-MB-231(Fig. 6E). The above results suggest that risk index may provide prognosis prediction and personalized treatment guidance for patients with TNBC.

## Discussion

TNBC is an aggressive subtype with significant heterogeneity and frequently develops resistance to treatment^17^. As per the current clinical guidelines, surgical resection, chemotherapy, and radiotherapy are the mainstay curative treatment options for TNBC patients^18,19^. Therefore, patients with TNBC generally have a poor prognosis, and there is an urgent need to explore the pathogenesis of TNBC further to improve clinical diagnosis and treatment. However, there are a few reports about the characteristics and the impact of NETs on patient survival in TNBC. The current study explored the characteristic of NET-related genes on the basis of bulk RNA-seq and scRNA-seq data, and established NET-related subtypes and a risk index that can be applied to clinical practice as a tool for prognosis and radiotherapy response prediction of TNBC patients. Further, it may improve the prognosis in TNBC patients by screening those who may benefit from treatment of Wnt signaling pathway inhibitors.

NETs are important components of the antimicrobial arms of neutrophils. Given the correct stimulus, neutrophils can extrude their nuclear DNA and reticular projects into the extracellular environment. Electron microscopy has shown that these reticuloDNA projections can be modified by a number of granular proteins, including NE, MPO, calguard, cathepsin G, proteinase 3, matrix metalloproteinase 9, and bactericidal/permeability-increasing protein^20,21^. During cancer growth, metastasis, and thrombosis, the excessive production of NETs and/or inadequate clearance may represent critical events. Therefore, therapeutic strategies that decrease abnormal NET production or facilitate NET degradation have potential clinical applications^22^. Several drugs can target NETs, and many are in development^23–25^. However, the formation and function of NETs in cancer tissue are yet to be fully elucidated. The characteristics of NET-related genes in tumor tissues need to be further analyzed to develop additional treatment modalities for TNBC.

NETs play an indispensable role in tumorigenesis and drug resistance. In pancreatic ductal carcinoma, interleukin (IL)-17 is highly expressed, and its recruitment of neutrophils triggers NETs to promote tumor multidrug resistance^26^. In high-grade gliomas, NETs induced by tumor-infiltrating neutrophils have also been shown to serve as oncogenic markers. In glioblastoma, NETs stimulate the NF-κB signaling pathway, thereby accelerating the secretion of IL-8 and further recruiting neutrophils. Tumor-infiltrating neutrophils mediate the formation of NETs through the PI3K/AKT/ROS axis, and through positive feedback, excessive NETs, in turn, promote the proliferation, migration, and invasion of cancer cells^27^. Our study showed that NET-related genes have genetic mutations and CNVs and were abnormally expressed in TNBC, indicating that NETs also play an important role in the normal mammary epithelial cell to proliferate abnormally and become cancerous. Besides, a novel NET-related subtype was identified with the help of TNBC-specific NET-related genes. The prognosis, functional enrichment analysis, and degree of tumor immune cell infiltration differed according in the two NET-related subtypes. Cluster A, associated with a better prognosis, was active in immune-related pathways and had a higher degree of immune cell infiltration. A previous report showed that a high extent of tumor cell infiltration is related to the positive response to adjuvant and neoadjuvant therapy in TNBC^28^. In other words, NET-related subtypes significantly distinguished TNBC patients with different survival outcomes, treatment responses, and immune microenvironment.

To further explore the value of NETs in the prognosis prediction and treatment of TNBC patients, the current study identified NET-DEGs between A and B subtype as potential biomarkers and constructed a risk index. The functional enrichment analyses (the GO and KEGG enrichment) showed that these potential biomarkers were relevant to immune-associated functions and signaling pathways, indicating that NETs play a key role in immunity, consistent with the results of the above studies. The communication between tumor cells and tumor microenvironment affects cellular biological processes through unequivocal signaling molecules, including ligands, receptors, metabolites, ions, and structural or secreted proteins. Inferring cell–cell interactions by integrating scRNA-seq data and gene expression with ligand-receptor information provides a new method to identify the underlying mechanisms of tumor progression^29–31^. The current study predicted the intercellular communication of hub DEGs identified through PPI network analysis based on scRNA data set and found that hub DEGs were mostly expressed in TNBC epithelial cells, T cells, and monocyte and PTPRC primarily played a vital role in TNBC via the GALECTIN signaling network, which was involved in the process of tumor immune evasion^32^. This finding provides a novel basis for further research on TNBC tumorigenesis.

Based on these potential biomarkers, a risk index and nomogram were established. In this study, the risk index exhibited reliable and sensitive performance in predicting the effectiveness of radiotherapy and chemotherapy in TNBC patients. These findings suggest a certain correlation between NETs and the effectiveness of chemo- and radiotherapy. Furthermore, previous studies have found that NETs could induce distant metastasis or recurrence of tumors by awakening dormant tumor cells and damaging endothelial cells to promote tumor cell infiltration^33,34^. In our research, survival analysis based on disease-free interval (DFI) showed a poorer prognosis for patients in the high-risk group compared to the low-risk group. This suggested that patients in the high-risk group are more susceptible to distant tumor metastasis or recurrence events.

To verify the results of our analysis, we further conducted out in vitro cell experiments. First, we calculated the risk score of 7 TNBC cell lines through the CCLE database, and MDA-MB-231 and BT549 with the highest and lowest risk scores were used in migration, invasion, wound healing, and colony formation assays. The experimental results showed that the MDA-MB-231 had higher level of malignancy, which was consistent with the results of bioinformatics analyses. A significant difference was found between high- and low-risk groups in their immune microenvironment and signature of the genome. In tumor immune infiltration analysis, we could find that immune cells (CD4^+^ T cell and M1 macrophage) with higher levels of infiltration in the low-risk group which play a role in consistent tumor growth in the tumor microenvironment^35,36^. TMB, which is consistent with PD-L1 expression, is a powerful prognostic biomarker for immune checkpoint blockade selection in different cancers^37^. BC with high TMB is more likely to benefit from PD-1 inhibitors^38^. In the present study, the low-risk group with better outcomes and therapy responses had more samples with gene mutations and higher TMB than the high-risk group, consistent with a previous study^39^.

Compared with other BC molecular subtypes, the treatment options for TNBC are few^40^, so it is particularly important to find new therapeutic drug targets for TNBC patients. According to the functional enrichment analysis of the high- and low-risk group, we found that TNBC patients in the low-risk group were significantly enriched in the Wnt and JAK/STAT signaling pathway. Surprisingly, patients in the low-risk group were more sensitive to treatment by Wnt signaling pathway inhibitors. Furthermore, the dose‐response growth curve of CCK-8 assay also validates the result that the cell viability of BT-549 with lower risk scores than those of MDA-MB-231 showed a significant decrease after adding XVA-939.

Despite the advantages of the current study, there are some limitations. First, the clinical application value of risk index was only validated in vitro; however, in vivo experiments must be carried out. Moreover, according to the above analyses, our conclusions provided a novel perspective for exploring the relationship between NET and TNBC, which should be proved by further experiments.

## Conclusions

NET plays a prominent role in TNBC. NET-related subtype based on NET-related genes clearly distinguishes patients with different characteristics. The risk index based on the NET-related potential biomarkers could provide a tool for predicting long-term prognosis and therapy responses in patients with TNBC; and, even more, identify potential beneficiaries of Wnt signaling pathway inhibitors. Furthermore, in vitro cell experiments also confirmed our findings.

## Materials and methods

### Data acquisition and processing

The baseline data of BC patients participating in the study has been listed in Table S1. The copy number, somatic mutation, bulk RNA sequencing (RNA-seq) data, and clinicopathological information of BC were gained from The Cancer Genome Atlas (TCGA) database (https://www.cancer.gov/ccg/research/genome-sequencing/tcga). The TCGA RNA-seq data were transformed into TPM format and designated as the TCGA-TNBC cohort for subsequent analysis. The microarray data of TNBC downloaded from Gene expression Omnibus (https://www.ncbi.nlm.nih.gov/geo/index.cgi) were GSE58812 and GSE135565. The microarray data were normalized using the "normalizeBetweenArrays" function in the R software. GSE58812 and GSE135565 were combined to form the GEO-TNBC cohort, and the “sva” R package^41^ exclusively addressed batch effects in these two GEO datasets. Moreover, in the process of constructing risk index, the data from TCGA-TNBC cohort were used for training cohort, meanwhile, the data from GEO-TNBC were utilized for testing cohort. Single-cell RNA-seq (scRNA-seq) data were downloaded from GSE161529 for the analysis of hub genes. A total of 136 neutrophil-related gene sets and NETosis-related gene sets, as the NET-related gene set, were obtained from previous study^42–45^ (Table S2).

### Somatic mutation and copy number variations (CNV) analysis

Waterfall plots, using the R package “maftools”^46^ were constructed to characterize the NET-related genes and tumor mutational burden (TMB). A comparison between diverse groups was carried out using the R package "ggpubr,"(https://cran.r-project.org/web/packages/ggpubr/index.html) and the correlation between risk index and TMB was visualized using boxplots and correlation plots. Besides, the frequency of CNV in NET-related genes was analyzed on the basis of the copy number data.

### Screening of TNBC-specific NET-related genes

The identification of TNBC-specific NET-related genes was based on differential expression and survival analysis, including univariate Cox regression and Kaplan–Meier (K–M) analyses. The R package "limma"^47^ was used to identify differentially expressed genes (DEGs) between TNBC and non-TNBC subtypes. The R packages “survival” (https://cran.r-project.org/web/packages/survival/index.html) was applied to survival analyses based on the above genes and K–M survival curves for significant DEGs (i.e., those with p-values < 0.05) were plotted. The optimal threshold for defining high and low expression of genes in the K–M analysis was determined using the "survminer" R package (https://CRAN.R-project.org/package=survminer) with the goal of including as many valuable NET-related genes as possible for subsequent analysis. R packages “ggplot2” (https://ggplot2.tidyverse.org) and “VennDiagram” (https://CRAN.R-project.org/package=VennDiagram) were used for plotting Venn diagrams. The prognostic and differential NET-related genes were obtained for subsequent analyses.

### Development of NET-related subtypes

R package “ConsensuClusterPlus”^48^ with parameters: reps = 50, pItem = 0.8, pFeature = 1, clusterAlg = “km,” distance = “euclidean,” and seed = 123,456 was used to separate patients into various subtypes in the integrated cohort. The “prcomp” function in the R software, and the R package “ggplot2” was used to simplify high-dimensional data for better visualization and analysis.

### Functional enrichment analysis

We carried out GSVA enrichment analysis visualized as a heat map constructed in the R package “GSVA”^49^. The subgroups identified by the R package "limma" were considered significant (adjusted p-value < 0.05). An analysis of GO functional enrichment and GO classification annotation of DEGs was performed using Gene Ontology (GO). The GO database (https://www.geneontology.org/) was used to determine the biological functions of the enriched GO terms. R package “clusterProfiler”^50^ was applied for the Kyoto Encyclopedia of Genes and Genomes (KEGG) signaling pathways and the GSEA enrichment analyses.

### Tumor immune cell infiltration: differential analyses

Scores from the single-sample gene set enrichment analysis were calculated using the “gsva” function in the R package “GSVA.” CIBERSORT algorithm^51^ was devoted to estimate the degree of immune infiltration in each sample. Correlation analysis between the risk signature and immune cell infiltration was performed using the R function “vioplot” (https://github.com/TomKellyGenetics/vioplot).

### Identification of hub NET-related DEGs

The Search Tool for the Retrieval of Interacting Genes/Proteins (STRING) online database (https://string-db.org/) was used to construct an interaction network between proteins, and the Cytoscape (version 3.8.1) software^52^ was used to visualize and analyze this result. Protein–protein interaction (PPI) networks were presented as figures, with nodes illustrating proteins and edges depicting associated interactions. The CytoHubba plugin^53^ in Cytoscape was used to identify core genes.

### Single-cell RNA sequencing data analysis

The R package “Seurat” (https://cran.r-project.org/web/packages/Seurat/index.html) was implemented to analyze scRNA-seq data. A Seurat object was created for the combined samples using the CreateSeuratObject function with parameters: min.cells = 3, min.features = 50. The percentage of mitochondrial reads was determined with PercentageFeatureSet function with pattern = “ˆmt-” parameter. Cells were filtered by nFeature (percent-mt (< 5) and & nFeature_RNA (> 50). Single-cell counts data were log-transformed using the NormalizeData function, with a scale_factor of 10,000. The first 20 principal components were clustered, and clusters with a resolution of 1 were identified. The t-stochastic neighboring embedding method (tSNE) was utilized to achieve the purpose of dimensionality reduction with the help of Seurat. The Seurat function “FindMarkers” and the Wilcox test were used to analyze the differential gene expression between clusters. According to DEGs between the clusters, cell types were confirmed. The Seurat toolkit “VlnPlot,” “DoHeatmap,” and “FeaturePlot” functions were used to generate violin, heat, and individual tSNE plots, respectively, for the given gene. After input of quality-controlled and normalized expression matrix, inference and analysis of cell–cell communication was performed using the R package “CellChat”^30^.

### Construction and validation of the risk index

The risk index, which could predict the outcome and cure responds of patients, was identified using machine learning algorithms (the adaptive least absolute shrinkage and selection operator (LASSO) based on the NET-related DEG (NET-DEG) expression matrix of TCGA-TNBC data (training cohort). The risk score for each patient was calculated using the following formula:

$$Risk\,score = \left( {GBP1P1 \times - 0.4559} \right) + \left( {MOXD1 \times - 0.2220} \right) + \left( {REEP6 \times 0.1293} \right)$$

The grouping of patients based on the median of the risk score (high- and low-risk groups). Parameters were tuned using the training cohort, and the validation cohort utilized the training cohort threshold to classify into high and low-risk groups. Between group differences in OS, gene expression, and outcomes were analyzed using K–M survival analysis, heat maps, and scatter plots, respectively. Time-dependent receiver operating characteristic (ROC) curves were used to estimate the predictive efficacy of the risk index and nomogram and drawn using the calculation procedure. Nomograms of the multivariable models were generated using the R package “rms” (https://CRAN.R-project.org/package=rms).

### Drug sensitivity analysis

Based on the Genomics of Drug Sensitivity in Cancer v2 (GDSC2) database (https://www.cancerrxgene.org/), we evaluated the half-maximal inhibitory concentration (IC50) of various drugs in the different risk score patients, by using the “oncopredict” algorithm^54^.

### TNBC cell lines and culture

MDA-MB-231 and BT-549 cells, were purchased from Procell (Wuhan, China). MDA-MB-231 was cultured in DMEM high glucose medium (Gibco, USA) supplemented with 10% fetal bovine serum (FBS) (Gibco), and 1% penicillin and streptomycin (P/S) at 37 °C in a moist incubator under 5% CO_2_. BT-549 was cultured in RPMI-1640 medium (Gibco) supplemented with 0.023 U/ml insulin, 10% FBS, and 1% P/S in a moist incubator at 37 °C under 5% CO_2_.

### Migration, invasion, and wound healing assay

Migration and invasion assays were performed in 24-well transwell chambers. TNBC cells were seeded into the upper chamber (8 mm pore size) with medium (DMEM high glucose or RPMI-1640), and the bottom chamber was filled with DMEM high glucose or RPMI-1640 containing 20% FBS. After 72 h, the cells on the lower surface of the filter were fixed and imaged, and five different fields of view was quantified using Image J software to get an average sum of cells. For the wound healing assay, cells were seeded into six-well plates at 5 × 10^5^ cells/well. Cell monolayer was scratched using a 200-μl pipette tip and washed with phosphate buffer solution to remove cell debris. Then the cells were cultured in serum-free DMEM high glucose or RPMI-1640 medium and each wound was imaged at 0 h, 24 h and 48 h, respectively after injury.

### Colony formation assay

Cells were planted and cultured in six-well plates at 1000cells/well, three replicate wells per experiment. After two weeks, the colonies were washed, fixed, stained, and recorded. The results were analyzed using Image J and Prism software.

### Cytotoxicity of Wnt signaling pathways inhibitor XVA-939

The number of cells was determined using the cell counting kit-8 (CCK-8, Solarbio, China) assay. MDA-MB-231 and BT-549 cells were resuspended in serum-free DMEM and RPMI-1640, respectively. The cell suspension (100 µL) was added to each well of the 96-well plate at a density of 8 × 10^3^ cells/well followed by 24-h incubation at 5% CO_2_ and 37 °C. 100 µL of the medium supplemented with 0, 0.01, 0.1, 0.5, 1, 2, 5, 10, and 20 µM of XVA-939 (Solarbio, China), respectively was added to each well and incubated for 24-h at 5% CO_2_ and 37 °C. Solution was removed from each well after incubation and the colorimetric solution (10 µL/well) was added into each well. After 2-h incubation, absorbance at 450 nm was evaluated.

### Statistical analysis

The differences between the two subtypes or the risk groups were evaluated using Wilcoxon rank-sum tests. The Kruskal–Wallis test and one-way analysis of variance were carried out to assess the differences among three or more groups. OS was compared among groups using log-rank test. Hazard ratios (HRs) were calculated, and independent risk factors were identified using univariate and multivariate Cox regression. All statistical analyses were conducted using R version 4.2.2. at a significance of p < 0.05.

### Supplementary Information

Supplementary Information.

## Data Availability

The datasets analyzed during the current study are available in the GEO (https://www.ncbi.nlm.nih.gov/geo/) and TCGA (https://portal.gdc.cancer.gov) repository, including GSE58812, GSE135565, GSE161529, and TCGA-TNBC.
