# Supplementary material for: Neutrophil extracellular trap-associated risk index for predicting outcomes and response to Wnt signaling inhibitors in triple-negative breast cancer
Source: Sci Rep. 2024 Feb 20;14:4232. doi: 10.1038/s41598-024-54888-y (PMC10879157; doi:10.1038/s41598-024-54888-y)
Supplement: Supplementary file 1 — Supplementary Information. [file 41598_2024_54888_MOESM1_ESM.pdf]

Supplementary Material

1 Supplementary Figures and Tables

1.1 Supplementary Figures

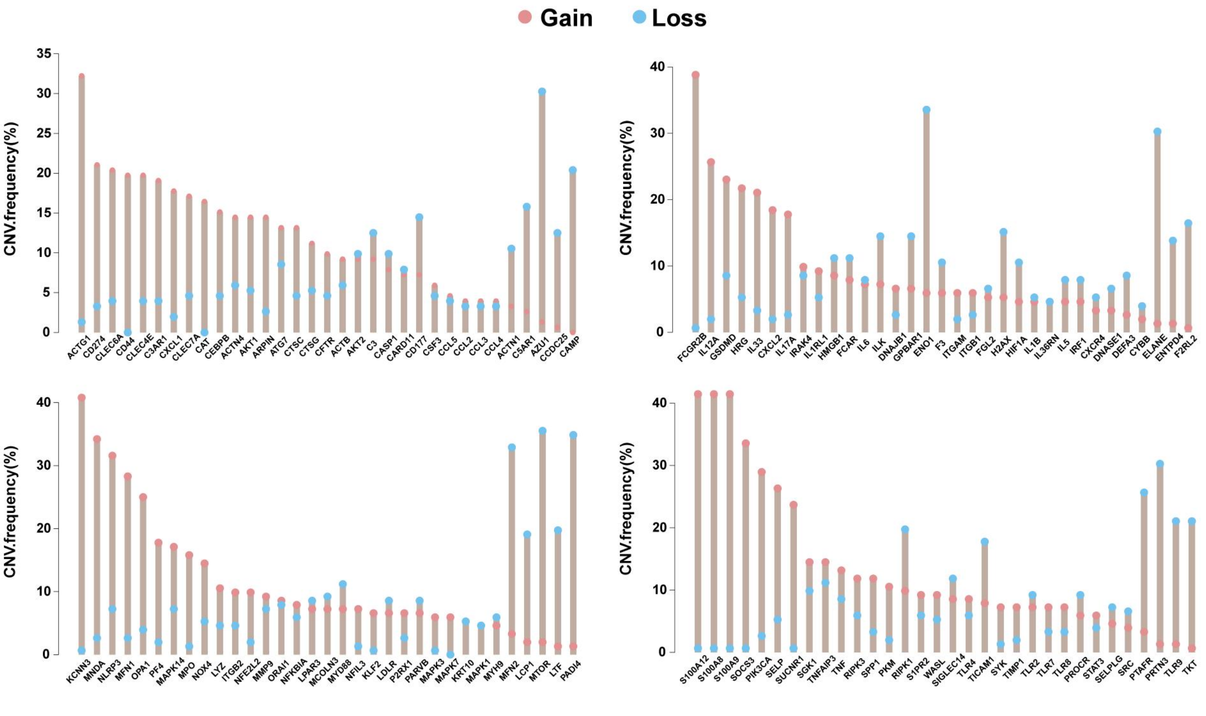

Figure S1. Copy number variation analysis of NET-related genes in TNBC.

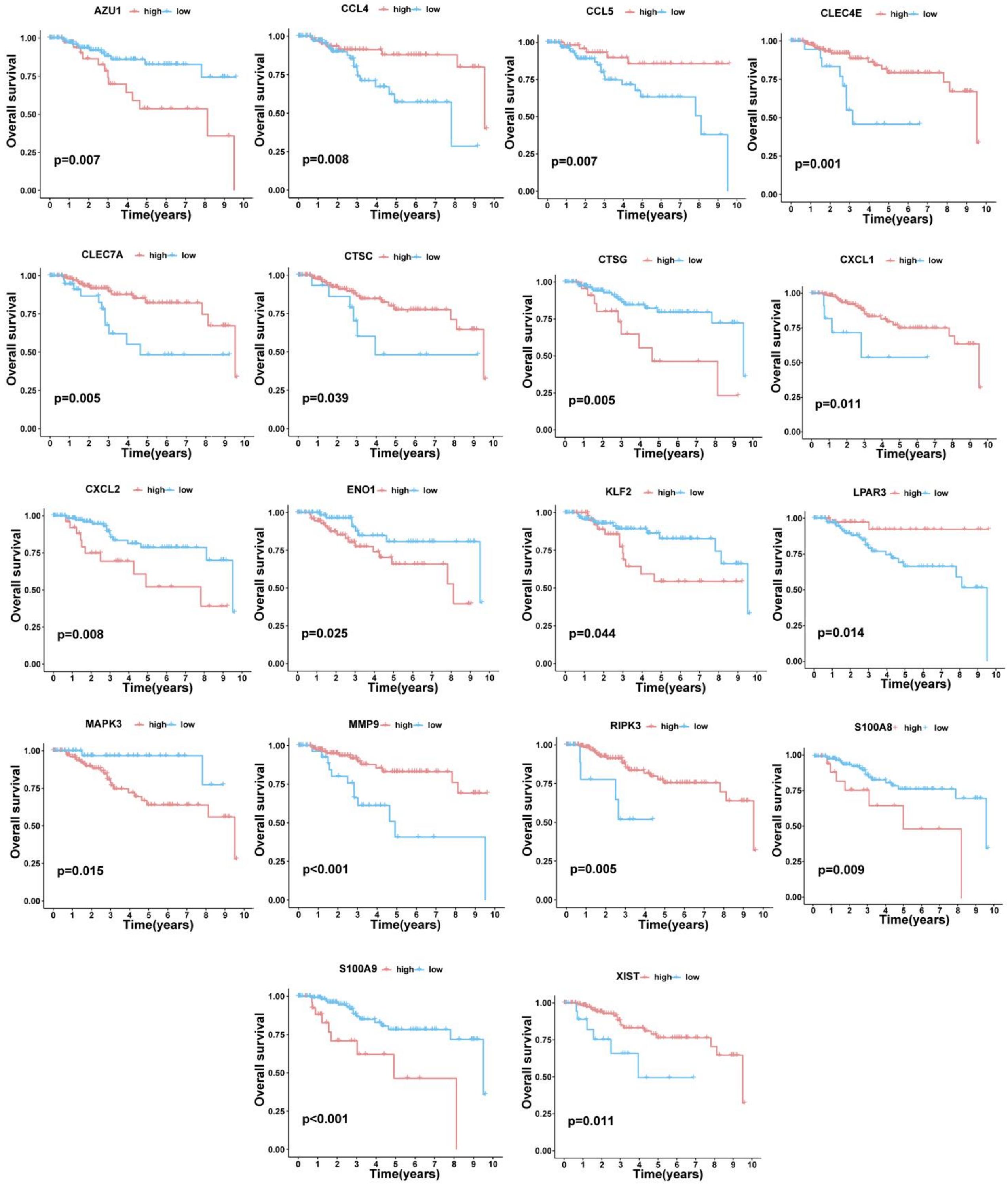

Figure S2. K–M survival curve of 18 NET-related genes with statistical significance in the analysis based on the TCGA-TNBC cohort.

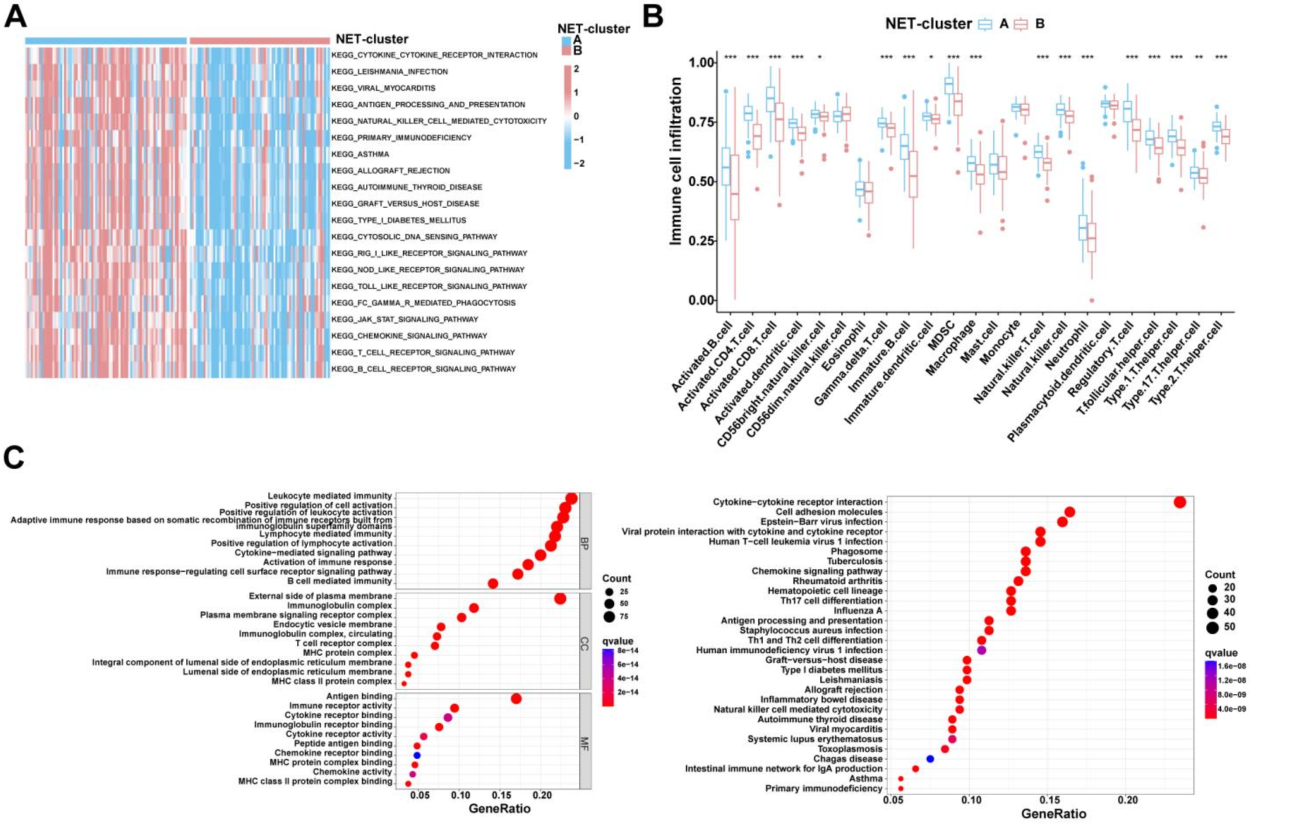

Figure S3. Functional enrichment analysis of differential genes between cluster A and cluster B

(A) Heatmap constructed in the R package “GSVA” displays the difference in functional enrichment between clusters A and B **(B)** The levels of tumor immune cell infiltration between clusters A and B. **(C)** GO and KEGG analysis of NET-DEGs (*, p<0.05; ***, p<0.001)

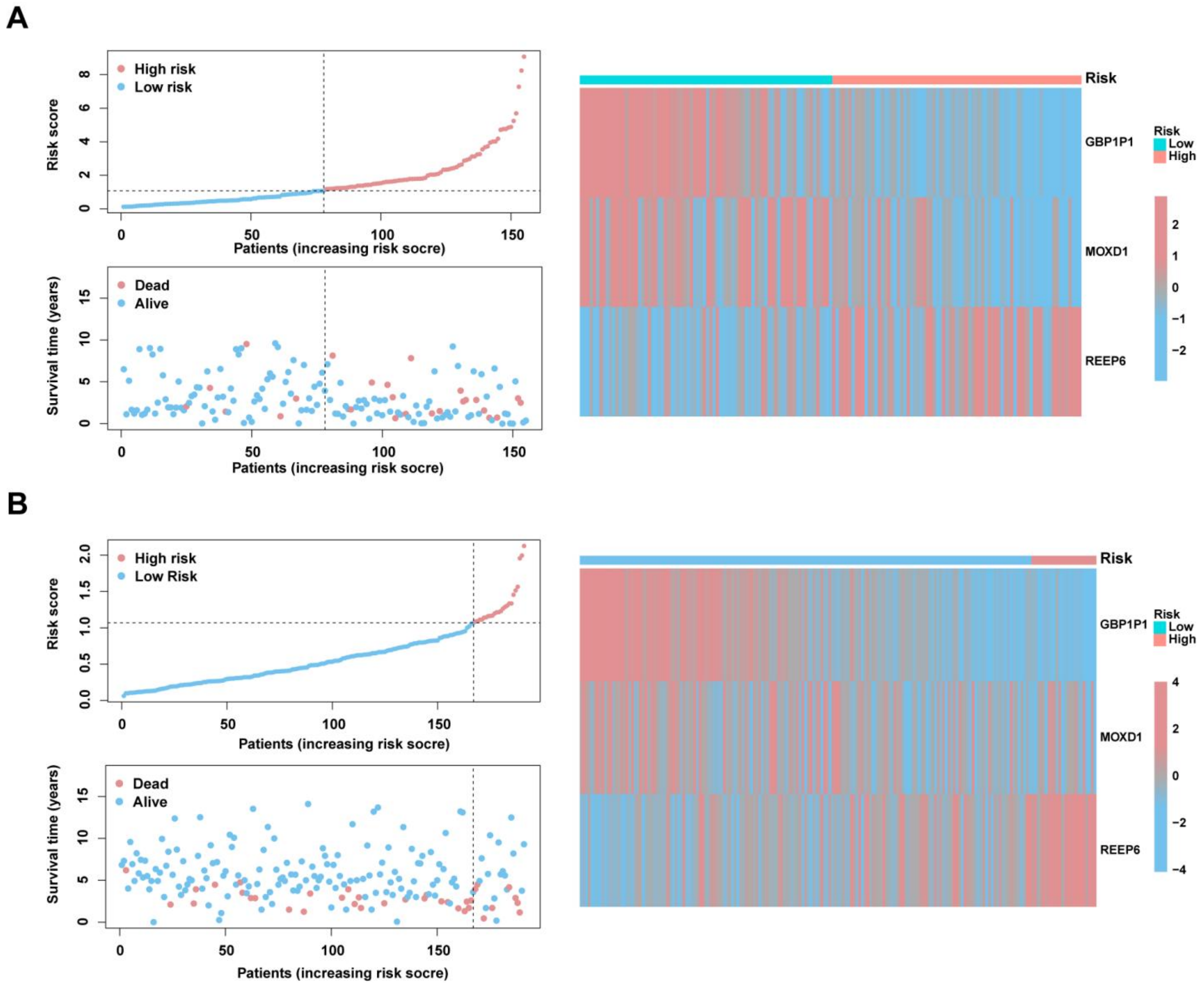

Figure S4. Scatter plots and heatmap showing the expression level of risk index-related genes in the **(A)** training cohort, **(B)** testing cohort

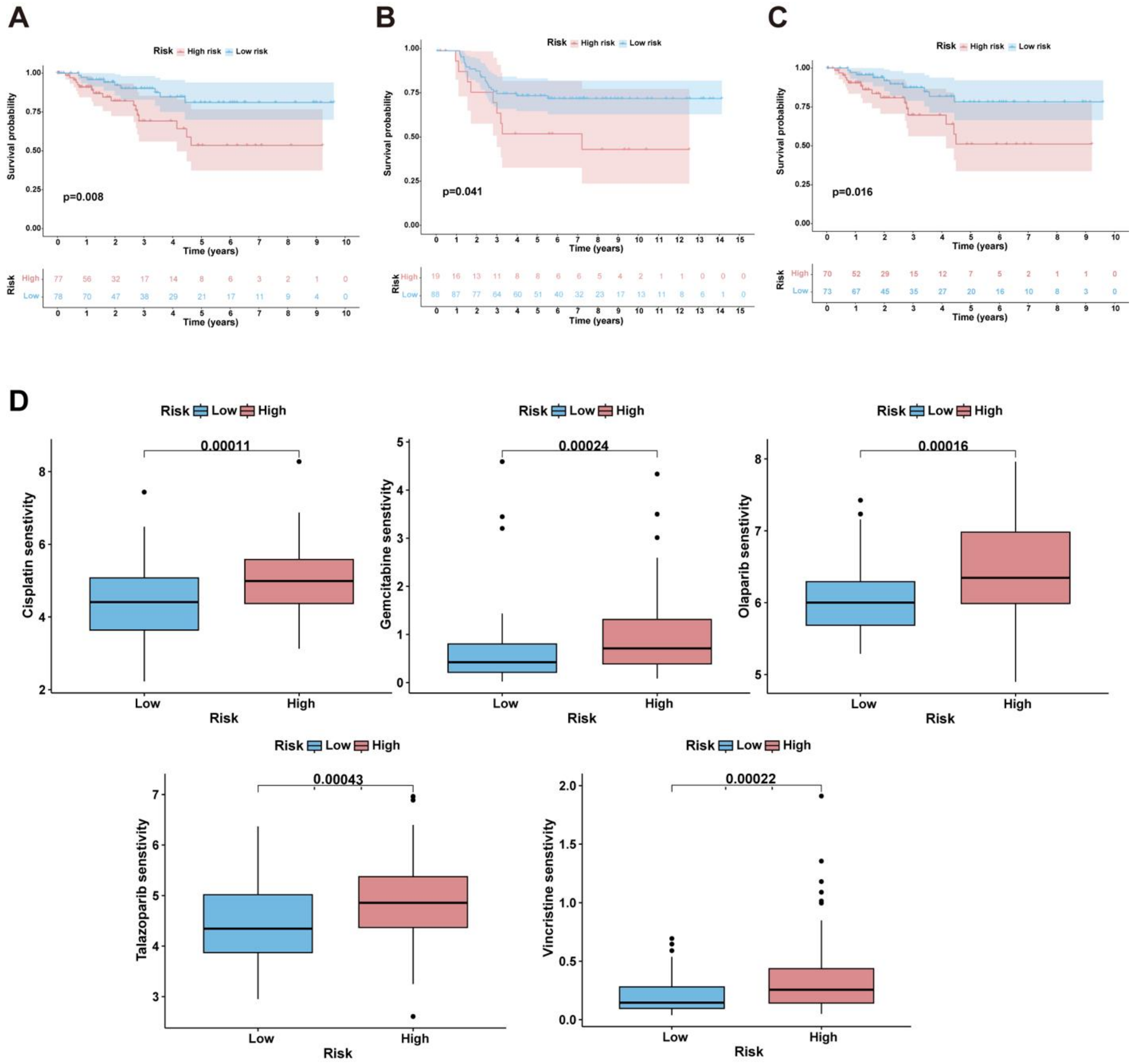

Figure S5. (A) The K–M survival curve analysis to assess the differences in the Disease-Free Interval (DFI) between the low-risk and high-risk groups, in the TCGA-TNBC cohort. **(B)** The K–M survival curve analysis to assess the differences in Metastasis-Free Survival (MFS) between the low-risk and high-risk groups, in the GSE58812 dataset. **(C)** The K–M survival curve analysis to assess the progression-free survival between patients with radiotherapy in the low-risk and high-risk groups. (**D**) The analysis of drug sensitivity of commonly used chemotherapy drugs in breast cancer.

1.2 Supplementary Tables

Variables	No. of Patients	Percentage (%)
**Menopause status**		
Pre-menopause	44	28.3
Post-menopause	96	61.9
Unknow	15	9.8
**Pathologic stage**		
I	28	18.1
II	98	63.2
III	24	15.5
IV	2	1.3
Unknown	3	1.9
**Pathologic T stage**		
T1	40	25.8
T2	95	61.3
T3	15	9.7
T4	5	3.2
**Pathologic N stage**		

N0	104	67.1
N1	30	19.3
N2	13	8.4
N3	8	5.2
**Pathologic M stage**		
M0	132	85.2
M1	2	1.3
Unknown	21	13.5
**Radiation therapy**		
No	62	40
Yes	81	52.3
Unknow	12	7.7
**Number of positive lymphnodes**		
0	96	61.9
1~3	25	16.1
4~9	13	8.4
⩾10	7	4.5
Unknow	14	9

Table S1. The baseline data of all patients with TNBC in the TCGA-TNBC cohort.

NET-related gene set							
ACTB	CCL2	CXCR4	HMGB1	KCNN3	MIR223	PF4	SPP1
ACTG1	CCL3	CYBB	HRG	KLF2	MMP9	PIK3CA	SRC
ACTN1	CCL4	DEFA3	IL12A	KRT10	MNDA	PKM	STAT3
ACTN4	CCL5	DNAJB1	IL17A	LCP1	MPO	PROCR	SUCNR1
AKT1	CD177	DNASE1	IL1B	LDLR	MTOR	PRTN3	SYK
AKT2	CD274	ELANE	IL1RL1	LPAR3	MYD88	PTAFR	TICAM1
ARPIN	CD44	ENO1	IL33	LTF	MYH9	RIPK1	TIMP1
ATG7	CEBPB	ENTPD4	IL36RN	LYZ	NFE2L2	RIPK3	TKT
AZU1	CFTR	F2RL2	IL5	MAPK1	NFIL3	S100A12	TLR2
C3	CLEC4E	F3	IL6	MAPK14	NFKBIA	S100A8	TLR4
C3AR1	CLEC6A	FCAR	IL8	MAPK3	NLRP3	S100A9	TLR7
C5AR1	CLEC7A	FCGR2B	ILK	MAPK7	NOX4	S1PR2	TLR8
CAMP	CSF3	FGL2	IRAK4	MCOLN3	OPA1	SELP	TLR9
CARD11	CTSC	GPBAR1	IRF1	MFN1	ORAI1	SELPLG	TNF
CASP1	CTSG	GSDMD	ITGAM	MFN2	P2RX1	SGK1	TNFAIP3
CAT	CXCL1	H2AX	ITGB1	MIR146A	PADI4	SIGLEC14	WASL
CCDC25	CXCL2	HIF1A	ITGB2	MIR21	PARVB	SOCS3	XIST

Table S2. 136 NET-related gene set.

CCL4	CD274	GBP4	DRAM1	TAGAP	HCP5	CCL7	KLHDC7B-DT
CLEC4E	CLEC7A	STAT1	WARS1	IDO1	MICB	EGFL6	NUP210
PDCD1LG2	GBP1	SAMD9L	HLA-B	TAP2	ARNTL2	IKZF3	LINC01871
CXCL10	APOL6	BATF2	IFNG	NLRC5	HLA-A	SEL1L3	AC022509.2
CTSS	ETV7	BCL2A1	C1S	GBP1P1	CCL18	SPIB	AC024940.1
OR2I1P	CXCR6	SOD2	PARP14	LINC02446	USP18	MOXD1	REEP6
GBP5	IFIH1	LAMP3	CGAS	CXCL9	CXCL13	HLA-H	
TAP1	CXCL11	GZMB	KLHDC7B	BTN3A3	C1R	NDUFB4P11	

Table S3. 62 prognostic NET-DEGs identified through univariate Cox regression analysis.
